# Evaluation of the Repatriation Process for Major Trauma Patients: Our Experience at Addenbrooke's Hospital, Cambridge University Hospitals NHS Foundation Trust

**DOI:** 10.7759/cureus.89151

**Published:** 2025-07-31

**Authors:** Ahmad W Mohamed, Masroor Ahmed, Mayank Kumar, Matija Krkovic

**Affiliations:** 1 Department of Trauma and Orthopedics, Addenbrooke's Hospital, Cambridge University Hospitals NHS Foundation Trust, Cambridge, GBR

**Keywords:** major trauma, major trauma centers, major trauma network, nhs england, repatriation, severely injured patients, trauma and orthopedics

## Abstract

Introduction: Major trauma patients succumb to significant injuries that require transfer to a major trauma center (MTC) for appropriate management. After the acute phase of treatment is over, the challenge arises when these patients require repatriation back to their local National Health Service hospitals for further rehabilitation. According to the “Major Trauma Clinical Network Specification,” one of the metrics is for patients to be repatriated to their local trauma unit within 48 hours of getting an accepting consultant. This study aims to evaluate the efficiency and feasibility of repatriating the patients within that timeframe.

Methodology: This was a retrospective study where we reviewed patients admitted to our Trauma and Orthopedics Department between January 2022 and June 2024. Medical records of patients were extracted through the hospital's IT system. Patients who were not local to our MTC and required repatriation were included in the study.

Results: A total of 224 patients were included in the study. Among them, 154 patients were repatriated to their local trauma unit. Only 31 patients (~14%) got repatriated within 48 hours of getting an accepting consultant. Seventy patients (31%) were discharged to their usual place of residence as they became medically and therapeutically fit for discharge before having an available bed. On average, four bed days per patient were lost during the repatriation process.

Conclusion: Repatriation is a resource-intensive process integral to major trauma care delivery. It helps patients in terms of functional recovery and psychological wellness, as they are closer to family and friends.

## Introduction

Injuries have been the leading cause of death around the world each year, resulting in nearly 8% of the deaths, around 4.4 million deaths worldwide [1]. Trauma is the leading cause of mortality under the age of 40 in the United Kingdom [2]. It also leads to increased morbidity and disability in the survivors [2]. There are estimates of 20,000 incidents of major trauma leading to 5,400 deaths every year [3]. Major trauma is defined as an Injury Severity Score of 15 or greater [4].

Major trauma patients sustain severe injuries that require management under a multidisciplinary team for optimal outcomes [5]. As a result, the National Health Service (NHS) established the trauma network in 2012, which follows a “hub and spoke” model. Each region has at least one major trauma center (MTC) that serves as the hub for severely injured trauma patients. This initiative aims to standardize care for these patients, which has improved their outcomes [4]. In established trauma networks, triage is the crucial initial step. After assessing the injuries and performing proper triage, if a patient meets the criteria for major trauma, they are transported to an MTC for definitive management rather than the nearest local trauma units [6]. Studies have demonstrated that management at MTCs, as opposed to local trauma units, is associated with better one-year outcomes, especially in patients with lower limb trauma [7].

The rehabilitation phase begins once the acute phase of injury management concludes and patients no longer require surgical or medical intervention. There is a need to transfer these stabilized patients back to their local hospitals for further rehabilitation. Repatriation has considerable functional and psychological effects on a patient, as proximity to family and friends positively influences their engagement in the rehabilitation process [8].

Given the importance of the repatriation process for patients and MTCs, we were interested in exploring it further and identifying reasons for delays. This study aims to evaluate the number of patients transferred through the major trauma network at Addenbrooke’s Hospital during a specified period, as well as the number of patients repatriated to their local trauma units/NHS trusts following the guidance and agreement of the major trauma networks.

## Materials and methods

Study design

This retrospective observational study was conducted in the Trauma and Orthopedics Department at Addenbrooke's Hospital, part of Cambridge University Hospitals NHS Foundation Trust, an MTC serving the East of England. The study period extended from January 2022 to June 2024. Approval for the study was obtained from the Institutional Audit and Research Committee. As this was a retrospective observational study involving no direct patient contact or intervention, formal patient consent was not required.

Study population

The study included patients transferred to Addenbrooke’s Hospital via the major trauma network for severe trauma and orthopedic injuries and were not local to Addenbrooke's. Eligibility required that patients had a named accepting consultant at their local trauma unit or NHS Trust for potential repatriation. Exclusion criteria included patients for whom Addenbrooke's Hospital was their local trauma unit and patients not admitted under the Trauma and Orthopedics Department as the primary clinical team.

Data collection

Patient data were extracted from the hospital’s electronic medical record system. The following variables were collected and recorded: gender, whether the patient was accepted for repatriation to a local trauma unit, time (in days) to repatriation, number of patients successfully repatriated, and number of patients not repatriated but instead got discharged to their usual place of residence.

Data analysis

All data were compiled and analyzed using IBM Statistical Package for the Social Sciences, version 25 (IBM Corp., Armonk, NY) and Microsoft Excel (Microsoft Corporation, Redmond, WA). Descriptive statistics were used to summarize patient characteristics and repatriation outcomes.

## Results

A total of 224 patients were included in this study. Out of these, 100 (44.6%) were women and 124 (55.4%) were men; 154 (68.75%) patients were successfully repatriated. Seventy (31.25%) patients were eventually discharged to their usual residence and were counted as failed repatriation. The number of patients successfully repatriated within 48 hours of receiving an accepting consultant was 31 (13.84%).

**Figure 1:**
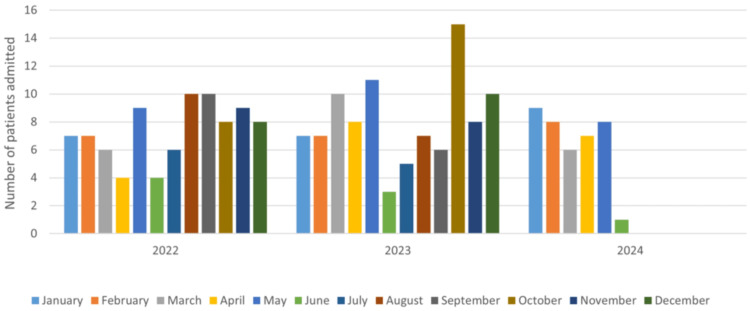
Number of admissions each month

The average number of days stayed in the hospital after getting an accepting consultant was eight days. The total length of hospital stay, defined as the time from admission to the hospital until discharge, was, on average, 21.4 days per patient. The number of patient admissions between September and February (inclusive) over the study period was 119 (53%), compared to 105 (47%) admissions between March and August. This variation was not statistically significant, as the p value was found to be 0.348 (p > 0.05). Furthermore, we stratified the data by the number of admissions in each year and found that 88 (39.3%) of the patients were admitted in 2022, 97 (43.3%) in 2023, and 39 (17.4%) in the first six months of 2024. The number of admissions each month is shown in Figure 1.

We extracted the patients’ notes to try and search for reasons for delays in repatriation. Unfortunately, extracting the patients’ notes had proven to be very difficult and unreliable. However, these are the most often recorded reasons that we were able to find: lack of bed availability at the patients’ respective local trauma units, closures of wards at those units due to isolation, the inability to accept patients isolated at our hospital because of infections like COVID-19, and delays in obtaining an accepting consultant due to difficulties in contacting the trauma and orthopedics team at the local trauma units.

## Discussion

In the United Kingdom, the introduction and expansion of MTCs led to the development of standardized protocols, such as those outlined in the "National Major Trauma Network-to-Network Repatriation Agreement." One key metric established in this agreement is that patients requiring repatriation should be allocated a bed at their local trauma unit within 48 hours of notification [9]. Regrettably, our evaluation revealed that only 13.84% of the patient cohort was repatriated within the specified timeframe. This finding underscores the need for further research to systematically stratify the causes of delay, enabling a comprehensive root cause analysis to implement effective corrective measures to prevent the implications mentioned later in this section.

A study by Safavi et al. evaluated the rate of successful patient repatriations from a tertiary center over the course of one year, including patients with various medical conditions beyond trauma. They found a 47.5% repatriation rate, but they did not specify the timeframe [10]. In contrast, our data demonstrated a higher repatriation success rate of 68.75%. It is important to note that while Safavi et al. reported an average tertiary hospital stay of 13 days, our cohort experienced a more extended average stay of 21.4 days. This discrepancy may reflect differences in patient demographics, injury severity and type, or variations in trauma system protocols.

Financial implications of repatriation delays were also considered, given their potential impact on healthcare resource management [11]. Our analysis revealed an average delay of eight days between the consultant's acceptance at the local trauma unit and the actual repatriation. This period resulted in an estimated loss of six bed days per patient. Based on data from Guest et al. [12], the cost per bed day is £586.59, translating to an approximate cost of £3,500 per patient. For our entire cohort of patients not repatriated within the 48-hour target, this delay corresponds to a total financial burden of approximately £675,500.

Delays in repatriation can harm both referring and receiving institutions, including limiting the number of beds available for new trauma admissions, in addition to the financial ones. If tertiary centers lack capacity due to repatriation delays, patients may experience prolonged waits at their local hospitals, possibly leading to the postponement of essential surgical interventions. Such delays can increase surgical complexity and adversely affect patient outcomes, including higher morbidity and mortality rates [13].

Psychosocial effects on patients and their support systems also warrant attention. Psychological distress associated with hospitalizations far from home may hinder recovery [14]. Yantzi et al. highlighted how travel distances for families can disrupt family dynamics and reduce the emotional support available to patients during hospitalization [15].

This study has several limitations. It was retrospective in design and, therefore, subject to bias. Repatriation destinations varied across hospitals, which may have introduced inconsistencies in delays. Additionally, the nature and severity of injuries differed among patients, potentially influencing rehabilitation needs and repatriation timelines, which may be relevant factors for future research.

## Conclusions

The repatriation of patients to their respective local trauma units still remains a daunting challenge for MTCs in the United Kingdom. However, this needs to be addressed, as delays have been shown to negatively impact patients, their families, and friends, as well as cause financial burdens at MTCs.

In conclusion, a viable solution would be to develop a system or pathway that ensures patients are repatriated within the specified 48-hour time limit. However, another proposed solution would also be to consider investment in rehabilitation facilities dedicated to such patients. The advantage of this approach would be that it not only helps clear out bed spaces but also improves patients' functional and psychological outcomes. We need to analyze this investment from a cost-effective perspective to see if it can alleviate the NHS's financial burden.
